# Purity or perversion? from taboo to fact: kindergarten teachers’ reflections on age-normal sexuality

**DOI:** 10.3389/fpsyg.2023.1212456

**Published:** 2023-07-19

**Authors:** Elisabeth Walsøe Lehn, Sobh Chahboun, Alexander Gamst Page

**Affiliations:** ^1^Institute of Pedagogy, Queen Maud University College, Trondheim, Norway; ^2^Department of Social Work, Norwegian University of Science and Technology, Trondheim, Norway

**Keywords:** Sexuality, child development, normality, kindergarten children, Taboo

## Abstract

Many educators and pedagogues around the world face challenging situations in their everyday work. Being caught off guard when children begin to explore their bodies and show curiosity about body parts and sexual issues is one of the most uncomfortable realities in the work of educating our children and can generate a series of worrying questions, such as, “Is this child* normal? Should I stop him/ her from masturbating? What should I tell him/her?. Although talking to children about body changes and sexual matters may seem strange or embarrassing, providing correct and age-appropriate information is one of the most important things kindergarten employees can do to ensure that children grow up protected, healthy and safe in their bodies. The current study is based on empirical evidence from focus group interviews with 18 kindergarten teachers from four different kindergartens. The aim is to provide a real overview regarding kindergarten employees’ experiences when it comes to their work with sexual development in small children. The findings show that sexuality is still a taboo even in western societies, as it is usually linked to abuse or pathological behavior. Additionally, key information about lack of focus on these topics in pedagogical educational programs is provided. Furthermore, the informants highlight the importance of knowledge and resources for them to feel in control and in confidence to face these challenges. Future directions and tips are provided to improve the educational field and ensure a healthy and balanced development which is after all part of all children’s rights.

## Introduction

Like all forms of human development, your child’s sexual formation begins at birth. This includes not only the physical changes that occur as children grow, but also the knowledge they learn, the beliefs they internalize, and the reactions adults have to the child’s early sexual explorations. Sexuality is innate and is a natural and expected part of human growth, stemming, among other things, from the drive for seeking closeness and love. Thus, it is a basic need that constitutes a facet of being human and cannot be separated from other aspects of life (WHO, 2006). That sexuality is innate and not learned may be inferred from the first signs of sexual reactions being observed in the fetal stage. Rather than young children experimenting as a reaction to internalizing adults’ feelings and understandings, they react to the pleasure experienced when exploring their bodies (Almås and Benestad, 2017). Such experiences are crucial for the ability to experience and take care of one’s own sexuality in a good way, and it is important that children get the opportunity to get to know their own bodies (Grünfeld and Almås, 2021). In the strategy for sexual health “Talk about it” (2017), it is stated that the foundations for positive sexual health are laid early, in which regard the kindergarten needs more knowledge of this aspect of development. Good sexual health is both a resource and a protective factor that promotes coping and quality of life (Ministry of Health and Care, 2016). The strategy is a clear political signal that helps to equate the sexual health of the youngest children with children’s general health, both physical and psychological.

There is a deep-rooted taboo surrounding discussions of sexuality in kindergarten (Kimerud, 2009; Skarpsno, 2013; Thorkildsen, 2015; Aasland, 2020), especially when it comes to topics such as sexual abuse and incest (Søftestad, 2018). This is likely connected to the limited linguistic and social tools we have for talking to young children about sexuality (Kimerud, 2017), which lead to children’s wonder and sexual exploration being curtailed (Friis, 2019). Friis (2019) argues that this could lead to children being robbed of an important part of their children’s culture and the right to privacy. Adults who have close relationships with children can do a lot of damage if they show discomfort when children express sexual feelings and display sexual behavior, thus imposing guilt on the child (Langfeldt, 2013). The experience of guilt, shame and fear can curtail the child’s normal development, creating a taboo around their sexuality, making it harder for them to distinguish fact from myth on the topic. This will also have consequences for how the child relates to their own sexuality later in life.

In the framework plan for kindergartens (Rammeplan for barnehagen– KD) (2017), the concept of sexuality is not explicitly invoked. However, the plan lays down clear guidelines for staff to contribute to children becoming familiar with and confident in their own bodies, thus gaining a positive perception of themselves and getting to know their own and others’ feelings. The staff must support children’s identity development and positive self-esteem. Children must learn to set limits for their own bodies and respect the limits of others, and their wonder and exploration must be met with recognition. The kindergarten must promote equality and equality regardless of gender, sexual orientation, gender identity and gender expression (KD, 2017). Even if the framework plan does not use the terms related to sexuality when referring to play, health, development or recognition, it is implicit, and should be reflected in the staff’s interpretation of the guidelines (Langfeldt, 2000; Bancroft, 2003; Træen, 2008; Kimerud, 2017).

The kindergarten has both a preventive and health-promoting responsibility, and should address all aspects of child development (KD, 2017). However, children’s sexuality is hardly visible in kindergarten research and education (Kimerud, 2017). This is problematic because the myths and taboos surrounding the topic can only be challenged through knowledge and education (Vildalen, 2014; Almås, 2020). Similarly, openness and knowledge on the part of the staff can give them an opportunity to reflect on how they can meet children’s sexual expression (Kimerud, 2017). By recognizing sexual exploration and play, staff can help children develop positive sexual health. This article explores how kindergarten teachers reflect together with colleagues about children’s sexuality and sexual exploration in kindergarten everyday life. The study is based on data obtained from five focus group interviews with 18 kindergarten teachers. The research questions are; how do kindergarten teachers relate to children’s sexuality and sexual exploration, and what reflections arise in the kindergarten teachers in conversations about children’s sexuality and sexual exploration.

### Historical review of children’s opportunities to explore and express their own sexuality

One potential limitation of this study is that it studies children indirectly, via the adults who take care of them, without children being able to contribute as informants. When the children themselves do not get the opportunity to give us the knowledge directly, history and research can contribute with different understandings of child sexuality. Epistemology or cognitive theory is a contribution to understanding how kindergarten teachers interpret today’s reality and what they perceive as valid knowledge, which further influences their practice. Kimerud (2009) outlines a historical retrospective from the 18th century, which shows that sexuality has gone from being seen as a great sin, via a cause of serious illness, to more liberal attitudes up until the 70s. In the 1970s, the focus on abuse prevention increased, which according to Jones (2003) helped to stop the liberalization process.

### The innocent and non-sexual child

Until the end of the 19th century, it was a common opinion in both medicine, psychology and pedagogy that sexuality first arose when people reached puberty (Skundberg, 2020). Rousseau (1991), known as the father of childhood in pedagogy, held that children needed to be shielded from the problems of the adult world, which has been highly influential until today (Almås and Benestad, 2017). He argued that the child was innocent and that sexuality would be an aberration of this innocence. For example, he argued that masturbation was a perversion, suggesting that it had to be prevented through correct upbringing, which was a prevailing view in the scientific community of the time. His view was particularly inspired by the doctor Tissot (2012) who similarly pathologized masturbation, suggesting that it sapped vital energies, and would lead to infertility. The pathologizing of sexuality and the shieling attitudes still remain, and lead to withholding information about sexuality from children (Løvereide, 2019). This is extremely difficult to change, because the idea of children as innocent and non-sexual has been constructed over so many years that, for many, sexuality becomes an invisible topic that neither is discussed nor problematized (Kimerud, 2017). Adults who protect and look after children express love and care, but seen in a critical light, they can also act oppressive and marginalizing.

### Different perspectives and views on child sexuality

The history of sexuality goes back a long way, but according to Bancroft, (2009), there have mainly been two epistemological perspectives that have dominated research into children’s sexuality. The traditional natural science where the idea of the innate authentic child sexuality which is as much as possible unaffected by external factors. Moreover, the postmodern understanding that considers childhood sexuality as culturally and socially created. Skundberg (2020) suggests that too much attention has been given to the origin of sexuality, arguing that the heterological perspective can contribute to us being able to assess sexuality based on the function and effect it has for the child rather than being concerned with whether it comes from within or from without.

A large number of studies also that parents’, educators’ and other professionals’ relationship and actual interpretation of young children’s sexual development, behavior and play is generally characterized by a hesitant, suspicious and uncertain attitude, despite a principled desire to recognize the natural sexuality of children (Balter et al., 2016; Brouskeli and Sapountzis, 2017; Davies et al., 2000; Heiman et al., 1998; Popovich et al., 2000; Stone et al., 2013). According to Martin (2014, p. 1637), child sexuality is understood alternately as natural, as a sign of abuse or as a warning of a “sex offender in the making,” and these conflicting possibilities make suspicion safer than harmlessness. Nevertheless, sexual behavior is not a sure indicator of abuse, because up to 40% of abused children show no sexual behavior at all (Friedrich et al., 2003).

### Homologous understanding of children’s sexuality

At the end of the 19th century, the attitude about the innocent child was challenged (Skundberg, 2020). Freud, who was a student of Charcot, is considered the discoverer of child sexuality, because of his three essays on sexual theory (1991[1905]). In his first essay, Infantile sexuality, he states that children have an innate sexuality. Freud, who had previously asserted that children’s sexual behavior was unhealthy tendencies and the result of artificial influence from the outside, was swayed by Moll’s research, which suggested a homologous approach, meaning that childhood and adult sexuality stemmed from the same biological and developmental drives (Sauerteig, 2012). Freud’s normalization of childhood sexuality must be nuanced according to Skundberg (2020) because Freud’s claimed that childhood sexuality had a future significance and was a preparation for adulthood. Freud claimed that children were polymorphically perverse, which means that they are attracted to both sexes, and that there was a deviation from the preferred heterosexual intercourse (Freud, 1991; Grünfeld and Svendsen, 2014).

Further suggested that children’s masturbation arose as a result of “seduction” from other children or adults (p.50) and if children were curious about other people’s bodies and showed off their bodies, it was one step on the way to seeing others as sexual objects, meaning that children had to learn to control their urges and desires (Skundberg, 2020). He considered most activities that created pleasure in the child as sexual, e.g., thumb sucking and going to the bathroom.

### Heterological understanding of children’s sexuality

Small children’s sexuality is both academically and politically recognized as a natural and healthy aspect of their play and development. Nevertheless, many studies show that parents and educators have a complicated and ambivalent relationship with children’s sexual behavior and play. The prevention of and vigilance for signs of abuse or harmful behavior means that age-normal behavior is perceived as disturbing or pathological, especially when it is associated with adult sexuality (Skundberg, 2020). Anchored in the child psychologist Charlotte Bühler’s critique of Freud’s sexual theory, and with concepts from Sauerteig's (2012) analysis of Bühler, it is argued that this is a question of epistemological understanding, and that the problem is partly that one interprets children’s sexuality as if it is triggered of, inspired by or created by impulses from adult sexuality.

Bühler’s research on child sexuality constitutes a break from earlier conceptualizations. She was critical of the homologous understanding and argued that childhood sexuality had nothing to do with adult sexuality. She saw children’s sexuality as fundamental, with intrinsic value and a natural part of the child’s development. Children’s sexual actions were not to be confused with adults, because their sexuality was mainly bodily characterized by exploration, sensory sensations and desire. She argued that most young children were easily distracted in their exploration, but that some children could become more concentrated and “hung up” on achieving the feeling of pleasure than others. In a heterological perspective, sexual play and behavior is therefore not a problem in itself, but must be covered by the same precautions, restrictions and risk assessments as all other play and activity (Sauerteig, 2012). In this way, Bühler is one of the few who consider children’s sexuality as an important and natural part of development and with intrinsic value for the child in the present (Skundberg, 2020). He argues that the heterological understanding is in line with prevailing ideals of seeing children as constructors of their own world of experience. Children’s sexual formation is an active testing process as a movement between free exploration and cultural socialization.

### Today’s discourse of child sexuality

Despite significant advancements in knowledge on the field, openness about sexuality has decreased since the 1960s (Langfeldt, 2000). A natural reason for this is probably that in the 1970s, sexual abuse of children was placed in a medical context that gave knowledge of how harmful it was. Thus, the positive relationship with sexuality was too vulnerable, so that we were unable to combine the problem of abuse with the positive and life-affirming sexuality Today, Norwegian legislation on violence and abuse against children is one of the strictest in the world (Aakvaag et al., 2016). In recent decades, various bodies have directed the spotlight on children’s right to a life free of violence and abuse. It has been a necessary and decisive boost in skills that must be worked on further (Emilsen and Lehn, 2020). Steine et al., (2016) concluded that it takes an average of 17.2 years before victims of sexual abuse come forward, and that some of the reasons for the delay were, among other things, that they were afraid of not being believed, of being shut down and that they lacked words and concepts. Through an increased focus on abuse prevention, the responsibility is placed on adults and the children are again seen as innocent and not – sexual. Kimerud (2017) argues that it can be challenging to accept that the youngest children have a sexuality that is significant. She questions whether the non-sexual thought creates a non-existent attitude toward child sexuality, that adults are unable to relate to it.

It may seem that children’s sexuality is often linked to abuse prevention in today’s Norwegian kindergartens. Skundberg (2020) points out that there is a need for research and theory on age-normal sexuality in order to be able to distinguish between the pathological sexuality, harmful sexual behavior and the sexuality that is age-normal, biologically and psychologically expected. He builds on several studies that show that educators look at children’s sexual development, behavior and play with an uncertain and suspicious attitude, even if they want to recognize the natural and age-normal child sexuality (Stone et al., 2013; Balter et al., 2016; Brouskeli and Sapountzis, 2017). Furthermore, a Danish study shows that the dominant discourse among the kindergarten staff and parents, on the subject of children’s “doctor games,” was characterized by boundary setting, vigilance for abuse and intentions to protect. While a small number saw child sexuality as healthy and natural (Leander et al., 2018). There are few scientific studies on the sexual development of the youngest children (Rademarkers et al., 2003; Kimerud, 2009). Within Norwegian educational research, there are few scientific studies related to the kindergarten context, there are, however, a few specialist books and bachelor’s and master’s theses. Students and educators express they have learned too little about children’s sexuality in education (Øverlien and Sogn, 2007; Kimerud, 2009; Island, 2009). A lack of thematization within kindergarten research and education will lead to the kindergarten teacher not being able to build on professional competence and have a reflective attitude (Kimerud, 2017). Children’s age-normal sexuality seems to be little thematized in kindergarten teacher training, management documents and research. In the “Escalation plan against violence and abuse against children” (2017–2021), however, everyone who works with children is held accountable and it is pointed out that children must receive age-appropriate training about body, identity and emotions (Ministry of Children and Equality, 2016). The plan is nevertheless in a context where sexuality is linked to abuse and prevention of the phenomenon. The strategy(…) “Talk about it,” on the other hand, is a clear political signal that equates the sexual health of the youngest children with children’s general health, both physical and psychological. The strategy is based on a heterological understanding that considers age-normal sexuality and children’s sexual exploration as an expected part of development. Good sexual health is established early and the kindergarten is an arena for recognizing children’s sexual expression and exploration. The strategy points out that good sexual health is a resource and protective factor that promotes quality of life and coping (Ministry of Health and Care, 2016). In the strategy, the youngest children have been given a place. Almås and Johannessen (2017) suggest that the plan clarifies the importance of sexual health from childhood to old age and there is a greater focus on the positive aspects of sexuality. Skilbred (2018) is critical of the fact that the strategy constructs sexuality as both a phenomenon that should benefit the individual’s health, but also focuses on the fact that it should benefit society as a whole. The consequence then is that sexuality ends up in the head, in the form of rationality and self-control, while the bodily and immediate experience is forgotten. In the framework plan for the kindergarten (2017), the concept of sexuality does not exist. The closest we can get is that the kindergarten must promote equality and equality regardless of gender, sexual orientation, gender identity and gender expression (KD, 2017). Several nevertheless take the floor that sexuality must still be implicit in the staff’s interpretation of the guidelines, despite the fact that the terms sexuality, sexual play, − health, − development or sexual recognition are absent (Langfeldt, 2000; Bancroft, 2003; Træen, 2008; Kimerud, 2017).

## Method and design

The current study is base on semi-structured focus group interviews where all the informants are kindergarten teachers and colleagues from four different kindergartens. The study is based on a social constructivist perspective which, in this context, means that the starting point is based on the fact that the kindergarten teacher’s understanding of the reality of children’s age-normal sexuality is shaped by the situation they find themselves in and the experiences they have, which in turn is linked to who they communicate with (Tjora, 2018).

The study is based on empirical evidence from focus group interviews with 18 kindergarten teachers, from four kindergartens. The informants were recruited through an e-mail request sent to practice kindergartens associated with a kindergarten teacher training program in Norway. The kindergartens in question are defined as large institutions, with four departments/bases or more and located in the same municipality. The selection of informants can be considered both strategic and random (Tjora, 2018). The kindergartens participating in the current study were randomly selected from a list of practice kindergartens, while the selection of informants was strategically chosen by the researcher to ensure answers to problems that focus on kindergarten teachers.

### Focus group interviews

Group interviews were a deliberate choice to bring out reflections because it is similar to way of collaboration that kindergarten staff often use in their daily work, which suggests that are used to it. Four group interviews were conducted with a total of 18 kindergarten teachers, with professional experience from 1.5 to 21 years in kindergarten. The interviews lasted 60–70 min and were audio recorded.

The group interviews were planned as semi-structured, where the goal was that they could reflect freely together, where the researcher collecting the data only acted as a mediator who brought the topic to the table. This was a conscious choice to bring out perspectives, contradictions and understandings during the group process. The advantage of group interviews for this study is that informants who are in dialog with each other can elicit a different type of information than if they were interviewed individually (Ringdal, 2018). The informants inspired each other and what was said had an “activating and mobilizing” effect on the others (Tjora, 2018). After the first interview, we nevertheless learned that we had to be clearer in distributing the word as the informants became eager and tended to talk over each other and side conversations arose. It then became demanding to take notes and follow along. In the next rounds of interviews, the researcher conducting the interview was a more active moderator (Tjora, 2018), which meant ensuring that everyone got the floor and the opportunity to complete their own reasoning. The participants were asked to take notes in order to be able to remember what they wanted to convey when the opportunity presented itself. The group interview form is suited to a social constructivist perspective because the participants both listen, think and express themselves as a group. However, this also makes the particularly challenging (Fern, 2001). As the participants listen to each other, and see the positive and negative reactions that others’ statements elicit, it is possible that their own perspectives shift. On the one hand, this is itself an example of socially constructed ideas, but on the other, there is a danger that the participants feign agreement. In order not to hinder good conversations, keywords were written down and the informants would be later asked to continue. In between, follow-up questions were asked such as, could you tell me a little more about it or what did you mean when you said etc.

The central topics were:

- Participants’ own base knowledge and relationship to children’s age-normal sexuality
- The place children’s age-normal sexuality and sexual health have in the participants’ kindergarten, management of educational processes
- The opportunities and challenges participants face in their management of age-normal sexuality as an educator
- How parental cooperation is experienced around age-normal sexuality

#### Analytical approach

Raw data material has been analyzed using a reflexive inductive approach where the themes were coded and classified. It will never be a pure induction as our preconceptions would constantly affect even when trying to identify data without predetermined theories (Richardson and Pierre, 2005).

#### Procedure

The interviews started with the researcher reading literature about children’s age-normal sexuality, but during the investigation, specialized research articles related to the kindergarten context were highlighted. Based on this, one of the main goals was to investigate how kindergarten teachers relate to children’s age-normal sexuality. The purpose was to develop an understanding of collected data that became something more than descriptions of practice. In such an interpretation, different relevant concepts can be related to the categories of the material (Thagaard, 2013). Therefore, the theoretical basis was developed during the analysis of the data collected. Based on the data base, we have endeavored to find a theory that can help frame and help discuss findings. Thagaard (2013) believes that various projects often have a center of gravity linked to the development of new theory or through further developing existing theory.

All the topics are relevant when the goal is to understand how kindergarten teachers experience their work with children’s age-normal sexuality. The themes represent different dimensions of the experience that the informants highlighted. The participants’ voices and use quotes to support them are emphasized.

#### Assessment of the method/credibility

It will not be possible to find a universal truth about this topic, but it is hoped that it can be a contribution to expanding the research field and that kindergarten teachers and students can make use of the results (Kvaale and Brinkmann, 2009).

#### Ethical considerations

By stopping the conversation midway, there may be a danger that some people did not get their full reasoning across as they might have done in an individual interview. Nevertheless, the conversations flowed well between the informants and they actively challenged each other. In the research process, there is a high probability that some findings have disappeared along the way. Bringing preconceived notions was controlled; full awareness about this has been taken into consideration during the entire process.

All the informants have given their consent and the audio recording was transcribed and deleted shortly after the interview in line with the Norwegian center for research data-NSD’s guidelines. The transcripts from the four groups of interviews constitute extensive empirical raw materials. Part of these have been focused on, analyzed and discussed in this current article.

## Result and discussion

### Sexuality is intimate and personal

The interviews started with a free association exercise, where the participants were asked to close their eyes for a few minutes before being encouraged to put into words what they immediately think about children’s age-normal sexuality. Almost all the participants expressed what was interpreted as negatively charged words and feelings: sensitive, difficult, embarrassing, shame, private and personal, challenging, abnormal, scary, serious and dark. The negatively charged words, and statements like “it’s not normal,” “you should not do this,” “embarrassing,” can indicate that they themselves carry personal experiences. This is borne out in the following dialogs from the conversations:

- “It’s a heavy topic because it’s so personal” (B -2)
- “Yes, my God, it’s not our sex life we are talking about in the break room exactly” (B -4)
- “It is difficult for me to accept what is normal in relation to the theory I have read, because it is not normal for me” (A − 1).
- “yes, it’s challenging for me to change my view on children’s exploration because it sits so well in my own body that you should not do that. Then I know at the same time that I am actually passing on the shame that was imposed on me” (A - 4)

The participants mainly start from the private and personal when they initially reflect freely on children’s sexuality, which may indicate that it can be difficult to relate to children’s sexuality without building on their own personal experiences. The ways in which these experiences played out, and the way they were met would have been decisive shaping the way in which the participants conceptualized sexuality as adults (Grünfeld and Almås, 2021). Subsequently, the adults’ reactions to their own experiences will be transmitted to the children in their care, as attachment to our closest caregivers is a contributing factor to how we manage our own and other people’s sexuality (Aasland, 2020). Other people’s feedback that creates a feeling that it is wrong to feel sexual feelings can lead to anxiety and guilt. A previous experience of shame is a bodily feeling that can influence thoughts and action patterns. The findings show that the participants are to a small extent able to distinguish between their own experiences and the role of pedagogues from the thoughts they immediately get when they have to express their thoughts about children’s age-normal sexuality.

Something that can be substantiated with Kimerud (2017), who argues that a lack of thematization in education means that kindergarten teachers will have challenges with reflecting and building on subject-related professional competence. Here a conflict can be glimpsed between a heterological understanding of knowledge and one’s own personal experiences which seem to be rooted in a homologous understanding which appears as an obstacle to recognizing children’s sexuality as a natural and expected part of development.

### Fear of stigma

During the interviews, it emerges that the informants experience situations where children’s sexual expression and exploration are not validated because the children are stopped, reprimanded or diverted. Assessments of themselves and feelings that they themselves are doing something wrong come out clearly, as well as the importance of what others think of them. This can be made concrete through selected quotes:

- “..children have always explored and masturbated, but we quickly divert and stop them.
- “yes, I sort of try to straighten things out a bit when others yell or get brusque” (B -1).
- “It becomes our responsibility that children are not violated, but how do we recognize the children without it being perceived as wrong or perverse in the ears of others?” (B-4)
- “..I am afraid of saying the wrong thing in view of other employees’ experiences from their own lives” (A-4) ”.
- “I have always been open, but have been met with a lot of skepticism. You are stigmatized, you who are concerned with sex, as it were. I’ve got that in my head. The problem is that the normal exploration is easily linked to sexual abuse, so I go around and try to correct when the others stop the children or are too strict. We have to meet them with recognition” (A − 3).
- “For me, this has become a natural part of pedagogy, I have knowledge of the topic that convinces me that this is important. It’s just as easy to get the others on board, it’s a lot about both emotions and judgment being challenged. I keep things like that to myself really” (C 1)

The way the conversations were interpreted, recognition of children’s sexuality is linked to a taboo and fear of stigmatization. As previously pointed out, it is a common perception that sexuality is a taboo subject in kindergarten (Skarpsno, 2013; Thorkildsen, 2015; Kimerud, 2017; Aasland, 2020). Fear of stigmatization can be a fundamental reason why the topic is not discussed or actively avoided. One expresses it like this: “I have never dared to bring it up as a leader, it is a sensitive topic” (B-3). Fear of being stigmatized can arise when attitudes are not reflected and seen in the light of theory and/or existing research. This may indicate that a “room” has not been created to talk together about practices related to children’s sexual expression, and that the staff lacks a common platform. Fear of being seen as sex-crazed and perverted can underpin previous interpretations regarding the fact that children’s sexuality is linked to the kindergarten teacher’s personal insecurities. Furthermore, it emerges that the staff do not talk together about practice, but mainly just practice. It seems that it is being communicated in the present when children’s sexual expression occurs, but that it is particularly based on spontaneous and unprofessional actions.

None of the informants has sexuality as a theme in the annual plan. Furthermore, it emerges that the topic is not the subject of professional reflection in formal meetings, but that communication occurs when children’s sexuality is brought up in daycare. Although many people think that it is normal, they are still afraid of other people’s reactions if they, for example, acknowledge children’s sexual play or answer questions from the children. This can be explained by the fact that the framework plan does not have sexuality explicitly expressed, it thus requires the educators to interpret the content (Bancroft, 2003; Kimerud, 2017).

If kindergarten teachers do not put the topic on the agenda for professional reflection, other employees probably will not either. However, and in line with the Norwegian framework plan for kindergarten education-KD (2017), it is the pedagogical leader who is responsible of implementing and leading the pedagogical work. Not all of the informants were pedagogical leaders, but the kindergarten teacher nevertheless plays a key role in meeting the requirements and intentions of the framework plan (KD, 2021).

### Is the concept of normality over-or underestimated? Just a tabu?

- “I’ve thought, why are you so concerned about this when somehow, the danger is that the others in the staff think I’m an abuser if I front this work” (B-2).
- “I have been afraid to be alone with children and enter into dialog with them if they ask, as a man you are extra exposed to suspicion, so I probably avoid quite a few situations” (D-1)
- “Before, I could look out the window and not think of any danger around, e.g., boys who had taken their pants down and were studying each other, but now I immediately think of abuse. Or…actually, I think about what the parents and other employees might think, if I do not intervene, and that there could be a lot of trouble. Then I become unsure of my own knowledge, even though I actually know a lot.” (A − 3)

The last two quotes show that the informants link sexuality to the problem of abuse, but also thoughts about their own leadership become more prominent. The kindergarten teacher’s knowledge of age-normal sexual development will have an impact on children’s right and opportunity for sexual play, − health, − development or sexual recognition.

A heterological understanding is made visible in the form of children’s sexual exploration being mentioned as self-evident in contrast to a homologous understanding which would not have recognized and allowed this. Nevertheless, it may seem that society’s focus on child abuse makes it difficult to distinguish between expected and age-normal sexual expressions and abuse issues. Something that is in line with the lack of focus in education and research (Kimerud, 2017).

### From individual thought processes to explicit reflection

Few of the informants have had education about age-normal sexuality in education. Several have taken part in courses with the theme of sexual abuse and where the focus has been on worrying and deviant behavior. Knowledge made them more confident, even so, responsibility can be great if not all employees integrate new knowledge. This is made visible in the following quote:

- “Yes.hmm, shifting the focus from abuse to the normal is also difficult. I think it’s pleasant, normal, exciting and perhaps a bit funny, while some in the staff group think that exploration and sexual play do not belong in the kindergarten. Disagreement and strong opinions can stop me. But if we are talking about abuse, no one disagrees about what we think about it like that” (A − 5)

During the interviews, all the groups touch on children’s masturbation and the informants expressed that this occurs regularly in the kindergarten. The informants explain that masturbation is natural and that they themselves believe that they have a clear relationship with this as part of the child’s development, yet it is experienced as challenging for the pedagogues. One of the informants’ experiences represents several of the informants’ experiences:

- “I do not make a big deal out of the children masturbating, I register it and move on. However, I had a girl who did it all the time, and when it prevented her playing and being with the other children, I felt like bringing it up at a department meeting. Only then I understood that not everyone thinks like me. It was difficult and unprofessional. Many attitudes emerged from some who were not professionally grounded, and that some did not think it was okay to masturbate at all. So even though I know this is natural and important, I do not get everyone on board. What I strive for is to have a professional discussion where we can free ourselves from our own and private perceptions. I’m afraid someone might interrupt the children and reprimand them if they masturbate.” (A2)

Several had similar experiences, and several told of situations where children were interrupted and reprimanded if they played exploratory sexual toys. The kindergarten teachers asserted that they themselves could personally look after children’s sexual expression and exploratory play in everyday life, but that the biggest challenge was getting the whole staff group involved. The informants described an uncertainty in relation to taking the lead and creating shared practices that ensure children’s right to explore, ask questions and play freely.

One of the informants has participated in a joint course day and development work in a kindergarten school she worked in previously. The topic was sexual abuse, but knowledge about children’s age-normal sexuality was a large part of the day. The informant says that she experienced a more unified understanding and enthusiasm in the staff group after the course. Informant C 2 says:

- “After the course, the boards took hold of the entire personnel group, we had several processes where we anchored the work in such a way that we got a professionally anchored basis from which to work. We elicited our own attitudes and experiences and then professional discussions. We arranged a parents’ meeting that only dealt with children’s sexuality with the same course instructor. It was useful and crucial to keep both us and the parents safe. We had a clear leader, so I think everyone understood that they have no choice, but some have to spend more time than others.” (C2)

The informant describes a development work where the board is a clear leader in the process. Her experience stands out, and provoked immediate reactions in the In the focus group interview in which she participated. It is probably an effect of the pedagogues being given the opportunity to reflect professionally on the topic, and gaining more faith in their own specialist knowledge.

One of the others spontaneously breaks in were:

- “But here we are, we just have to start talking about it. Here you are sitting on valuable knowledge without me knowing about it. I hear that you are also concerned with the topic, but that you find it difficult to deal with it. We have each other. I already know that I am more motivated to lead my group now, I am not as alone as I have felt. And then we must have a clear director who legitimizes” (C1)

The quote above represents well what happened in the other group discussions as well. The participants became very enthusiastic during the focus group interview and many thoughts emerged about how they could best develop their management related to the place of sexuality in the kindergarten. Here the findings show that the participants call for the place of sexuality in the framework plan. One of the participants expressed the following:

- “Legislation and the framework plan are good support for me, it’s not like I’m concerned with this and that, but it is determined and anchored. Nevertheless, it would have been a good help if children’s sexuality had been made clear. Children’s sexuality is not the same as ours, but it is not so easy to understand, we mix it up and then it is easily associated with abuse.” (A4)

At the same time, several participants state that the framework plan that came out in 2017 is clearer on the preventive and health-promoting perspective than the framework plan from 2017. They say that they can find more arguments for the work under the subject areas; body, health and movement and equality, as well as in the value base and related to coping with life. Furthermore, the participants suggest that themes such as the body and sexuality must be anchored as a theme in the annual plan for the kindergarten, and that a progression plan is necessary. Clearer guidelines, literature and research emerge as a need, but most participants place responsibility on the boards, who are expected to take the lead in putting the topic on the agenda together with the management team.

- “We see that knowledge in itself is of little value, we know a lot about this topic when we just sit down and talk about it. But we must stand together and we must discuss and reflect aloud together, we must professionally anchor the work in order to rid ourselves of myths, taboos and attitudes that each individual carries with them in the face of this topic” (D-2).
- “Yes, I think we can actually uncover more people who are exposed to abuse if we manage to recognize children’s sexuality and exploration as a natural part of their development. How can we establish trust if we stop and prevent everyone” (D -4).

## Conclusion

Based on the lack of focus in education (Kimerud, 2017) and the fact that sexuality as part of children’s development is not explicitly expressed in the Framework Plan (2017), it may appear that educators are in a cross-pressure between the desire to recognize children’s sexual expression and the fear of being stigmatized.

Both higher education and pedagogues in kindergartens also lack relevant and sufficient research and literature that can challenge myths and taboos (Almås, 2020), as well as expand theoretical understanding and underpin the importance of facilitating children’s sexual health, which is pointed out in the strategy, “Talk about it” (Ministry of Health and Care, 2016).

We also see that a focus on sexual abuse of children and the kindergarten’s responsibility to prevent and detect (Emilsen and Lehn, 2020) can dominate the kindergarten teacher’s practice, when they lack theoretical knowledge of age-normal sexual development. Through an increased focus on the prevention of sexual abuse, the child will easily be seen as not - sexual, i.e., a homologous view which, according to Skundberg (2020), dominated in the 20th century.

Through the analysis of data from the group interview, an effect of sitting together with other pedagogues is made visible. The participants discover that by having time and space to discuss and share their experiences, they become more confident in their own knowledge. This is in line with claims from several researchers who point out that there is a deeply rooted taboo in the nursery, as well as a lack of language and knowledge about age-normal sexual development in young children (Skarpsno, 2013; Thorkildsen, 2015; Kimerud, 2017; Aasland, 2020).

## Future research

Further research will be necessary to find out how kindergarten teacher training can facilitate the pedagogues to gain more theoretical knowledge, and help to interpret the content of the framework plan (KD, 2017) which focuses both on sexual orientation, play, health and not least recognition. It will be equally important that education facilitates the students to have the opportunity to discuss and reflect together. Discussions based on cases will be able to enhance both the ability to take different perspectives and increase the confidence to be able to defend pedagogical measures when they go out as leaders in the nursery school.

Students will need more teaching to distinguish between a homologous and a heterological understanding, so that they can work both health-promoting and preventive, as the framework plan (KS, 2017) points out. Children have the right to be met with openness and recognition also in this part of their development, which according to Skundberg (2020) is about prevailing ideals where children are seen as constructors of their own life world.

## Data availability statement

The raw data supporting the conclusions of this article will be made available by the authors, without undue reservation.

## Ethics statement

The studies involving human participants were reviewed and approved by Norwegian center for research data. The patients/participants provided their written informed consent to participate in this study.

## Author contributions

EL collected and analized the data. All authors contributed to the article and approved the submitted version.

## Conflict of interest

The authors declare that the research was conducted in the absence of any commercial or financial relationships that could be construed as a potential conflict of interest.

## Publisher’s note

All claims expressed in this article are solely those of the authors and do not necessarily represent those of their affiliated organizations, or those of the publisher, the editors and the reviewers. Any product that may be evaluated in this article, or claim that may be made by its manufacturer, is not guaranteed or endorsed by the publisher.
